# Microbial Hyaluronidases: From Obscure Virulence Factors to Promising Therapeutic Targets

**DOI:** 10.3390/biom16040516

**Published:** 2026-03-31

**Authors:** Hannah A. Nonoguchi, George Y. Liu, Irshad A. Hajam

**Affiliations:** 1 Department of Pediatrics, University of California, San Diego, CA 92093, USA; hnonoguc@health.ucsd.edu; 2 Division of Infectious Diseases, Rady Children’s Hospital, San Diego, CA 92123, USA

**Keywords:** β-elimination lyases, hyaluronan, hyaluronidases, microbes, virulence factors

## Abstract

Hyaluronan (HA) is a major extracellular matrix glycosaminoglycan essential for tissue integrity, immune homeostasis, and host defense. Many microbial pathogens exploit host HA by producing hyaluronidases (Hyls), enzymes that degrade HA to promote tissue invasion, nutrient acquisition, immune modulation, and biofilm formation. Unlike mammalian Hyls, microbial Hyls predominantly function as β-elimination lyases, generating unsaturated disaccharides and oligosaccharides with distinct biological activities. Recent mechanistic and structural insights reveal that distinct microbial Hyl variants uniquely shape host–microbe interactions and disease outcomes. This review focuses on microbial Hyls, specifically bacterial Hyls, emphasizing their roles in host immune regulation and inflammatory diseases, particularly in *Cutibacterium acnes*-mediated acne pathogenesis. We also discuss emerging therapeutic strategies targeting the HA-Hyl axis to modulate inflammation, highlighting their potential as a foundation for novel human therapeutics.

## 1. Introduction 

Hyaluronan (HA), also known as hyaluronic acid or hyaluronate, is a hydrophilic, non-sulfated, unbranched glycosaminoglycan (GAG) composed of repeating disaccharide units of D-glucuronic acid (D-GlcA) and N-acetyl-D-glucosamine (GlcNAc) linked by alternating β-1,4 and β-1,3 glycosidic bonds [1,2]. HA is a ubiquitous component of the vertebrate extracellular matrix (ECM), where it contributes to tissue structure, immune regulation, and wound healing [3,4,5,6]. It is synthesized by a variety of cell types, including fibroblasts, keratinocytes, endothelial cells, and smooth muscle cells, and is particularly abundant in the skin, synovial fluid, connective tissues, and lymphoid organs [1,7]. In mammalian cells, HA is primarily synthesized at the plasma membrane by three distinct isoforms of hyaluronan synthase, namely HAS1, HAS2, and HAS3, which differ both structurally and functionally [2,8]. HAS1 and HAS2 preferentially synthesize high-molecular-weight HA (HMW-HA; ~2000 to 6000 kDa), whereas HAS3 predominantly generates lower-molecular-weight HA (LMW-HA; ~100 to 1000 kDa) [9]. 

**Figure 1:**
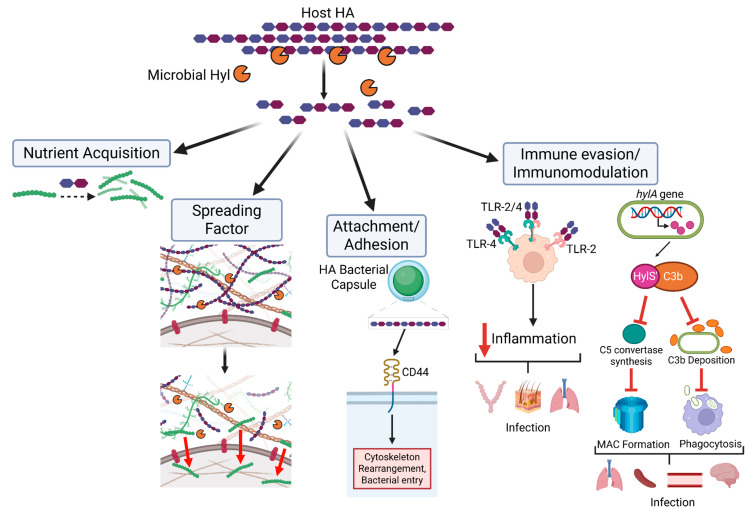
Bacterial manipulation of host HA to drive colonization and infection. Pathogens exploit HA through multiple mechanisms, including nutrient acquisition via HA catabolism; degradation of HA as a “spreading factor” to facilitate tissue dissemination; HA-mediated attachment and adhesion to host cell surface receptors, activating intracellular signaling pathways; and immune evasion through modulation of innate immune responses, such as blocking TLR2/4 macrophage signaling and inhibiting complement activation across diverse host tissues. HA: Hyaluronan; Hyl: hyaluronidase; hylA gene: *S. suis* hyaluronan gene; HylS’: *S. suis* Hyl fragment; MAC: membrane attack complex. Created in BioRender. Nonoguchi, H. (2026) https://BioRender.com/kkqow3a (accessed on 6 February 2026).

HA serves as a dynamic signaling molecule by interacting with multiple host receptors to regulate immunity and tissue homeostasis (Figure 1). While HA-TLR2/4 interactions represent a key axis of inflammatory regulation [10], they account for only a fraction of HA’s immunological influence. HA also signals through other receptors, including CD44 [11,12], which promotes tissue homeostasis and modulates inflammation via NF-κB and phosphorylation cascades; RHAMM, which regulates cell motility, inflammation, and cancer through protein tyrosine phosphorylation and ERK signaling [12]; LYVE-1, which facilitates immune cell entry into lymphatics [13]; and HARE, which is responsible for HA clearance from the circulation [14,15,16].

The biological activity of HA is largely size-dependent. Under homeostatic conditions, intact HA restricts the infiltration of inflammatory cells; however, Hyl-mediated degradation increases tissue permeability, facilitates the recruitment of innate immune cells, and thereby exacerbates inflammation [11,17,18]. HMW-HA is generally associated with anti-inflammatory, immunoregulatory, and tissue-protective roles [10,19], whereas LMW-HA fragments function as danger-associated molecular patterns (DAMPs) that activate innate immune cells to trigger inflammatory cytokine production [9,10,20,21,22,23,24]. These LMW-HA fragments are generated during tissue injury through oxidative stress or the activity of mammalian or bacterial hyaluronidases (Hyls) [8,24,25,26]. Given the size-dependence of HA biological functions, the activity of HA-synthesizing enzymes is tightly regulated through transcriptional control, substrate availability, post-translational modifications, and external cues such as cytokines, growth factors, and microbial components [27,28,29,30,31,32]. This multilayered regulation enables cells to dynamically modulate HA production in response to physiological and pathological conditions, underscoring HA as a versatile regulator of tissue homeostasis.

Mammalian hosts are colonized by diverse microbes that secrete Hyls, most of which are polysaccharide lyases that degrade HA into fragments of varying sizes [2,24,33]. Although mammalian and microbial Hyls act on the same substrate, they are fundamentally distinct in their structural organization, catalytic mechanisms, and biological functions [2,8,24,34]. These differences reflect their divergent evolutionary origins and underlie their contrasting roles in tissue homeostasis versus host–microbe interactions. Recent reviews published over the past several years have extensively described the biochemical properties of HA, its receptors, and the physiological functions of mammalian Hyls in tissue remodeling and inflammation. However, many of these studies have largely focused on host HA metabolism and signaling pathways, with comparatively limited attention to the diversity, regulation, and functional consequences of microbial Hyls in host–microbe interactions. In particular, the contribution of microbial Hyl variants to disease progression and immune modulation remains insufficiently explored. Moreover, the role of Hyls in dermatological conditions such as acne has received little dedicated attention in previous reviews. In this review, we expand upon the existing literature on microbial Hyls, including their regulatory mechanisms, functional diversity, and their dual roles in promoting either pathogenicity or host-microbial homeostasis, especially in the context of acne. Additionally, we discuss emerging therapeutic strategies aimed at modulating mammalian and microbial Hyl activity to influence HA-dependent processes in health and disease.

## 2. Mammalian and Microbial Hyls: Classification, Function, and Biological Distribution

Hyls are enzymes that degrade HA and are ubiquitously distributed across the biological spectrum. They are present in both higher organisms and microorganisms and have been extensively studied for their roles in physiology and disease. Structurally and functionally, Hyls can be classified into three principal categories, as proposed by Meyer et al. (1971), based on substrate specificity and catalytic mechanism [35]. Hyaluronoglucosaminidase (EC 3.2.1.35) is a glycosidase hydrolase predominantly found in mammals and venoms of various insects and snakes. They hydrolyze β-1,4-glycosidic bonds in HA, producing tetra- and hexasaccharides with N-acetylglucosamine at the reducing end [8,35,36,37,38]. Hyalurono-glucosidase (EC 3.2.1.36), derived from leech salivary enzymes, selectively cleaves HA via the β-1,3-glycosidic bond, yielding tetrasaccharides with glucuronic acid at the reducing end [39,40]. Compared to the other two categories, polysaccharide lyases (PL-class) are microbial Hyls that cleave the HA β-1,4-glycosidic bonds via a β-elimination mechanism, producing fragments such as disaccharides and larger oligosaccharides with a non-reducing unsaturated end [24,34,41]. This catalytic dichotomy between mammalian and microbial Hyls reflects fundamentally distinct HA degradation pathways and the generation of chemically distinct products. In humans, six Hyl-related genes have been identified, including Hyl1, Hyl2, Hyl3, Hyl4, PH20 (SPAM1), and HylP1, which share approximately 33–44% sequence homology [2]. Among these, Hyl1, Hyl2, and PH20 exhibit the greatest enzymatic activity and biological significance, whereas Hyl3 has been proposed to function as a non-enzymatic regulator of Hyl1, but its catalytic activity has not been clearly established [8]. Hyl4 has no Hyl activity, and HylP1 is considered a pseudogene without confirmed catalytic activity [42]. Human Hyls are expressed across multiple tissues and adopt a conserved TIM-barrel-like fold that enables regulated HA catabolism at the cell surface, in endosomes, or within lysosomes [43,44,45,46,47]. Mechanistically, they act as hydrolases that cleave the β-1,4 glycosidic bonds in HA via acid-base catalysis, generating saturated oligosaccharides involved in diverse biological processes, including HA turnover, tissue homeostasis, cell signaling, migration, proliferation, and immunomodulation [19,34,48,49,50]. In addition to classical Hyls in humans, other HA-binding proteins, including CEMIP (KIAA1199; cell migration-inducing protein) and TMEM2 (transmembrane protein 2), contribute to HA depolymerization into intermediate-sized fragments (~5–100 kDa) [2]. TMEM2 is a transmembrane protein that has been reported to possess intrinsic Hyl activity at the cell surface in a calcium ion-dependent manner. Although CEMIP is essential for HA degradation and promotes HA depolymerization, it remains unclear whether CEMIP itself catalyzes HA cleavage or instead facilitates HA processing indirectly through associated factors. A study by Yoshino and colleagues reported on the indispensable role of CEMIP in HA degradation in the skin dermis, as knockdown of this gene in skin fibroblasts completely suppressed HA catabolism [51]. This study was supported by other studies where CEMIP-deficient mice accumulated HMW-HA in the brain [52]. Although CEMIP has been implicated in HA degradation, its catalytic mechanism remains incompletely defined and differs from that of classical human Hyls. Human Hyls such as Hyl1 and Hyl2 degrade HA through a well-characterized hydrolytic mechanism that cleaves the β-1,4 glycosidic bond via acid–base catalysis, generating smaller HA fragments. In contrast, current evidence suggests that CEMIP may promote HA degradation through a distinct process that does not fully resemble the classical hydrolytic pathway and may involve lytic or non-canonical mechanisms associated with intracellular trafficking and endosomal processing of HA [53,54,55]. These mechanistic differences highlight that CEMIP-mediated HA degradation likely represents a functionally distinct pathway from the enzymatic hydrolysis carried out by canonical Hyls, an area that remains under active investigation. Given the biological importance of mammalian Hyls in regulating HA turnover and tissue homeostasis, exogenous Hyls have been widely developed for clinical and laboratory use. Currently, most marketed Hyls are derived from mammalian testis, with bovine and ovine testicular extracts exhibiting particularly high enzymatic activity [56]. Among these, bovine testicular Hyl (BTH) has been the most widely used clinically, despite sharing only modest sequence homology (22.9–25.2%) with human Hyls [45]. While mammalian Hyls primarily function in normal ECM turnover and HA catabolism, microbial Hyls often function as virulence factors that facilitate tissue invasion, nutrient acquisition, and immune manipulation [10,24,33,57]. The classification of Hyls proposed by Karl Meyer has historically served as an important framework for distinguishing HA-degrading enzymes based on their biochemical activity and degradation products. However, advances in structural biology and genomics have led to more refined classification systems. Contemporary resources such as the CAZy database categorize HA-degrading enzymes according to sequence similarity, structural architecture, and catalytic mechanisms. Within this framework, many microbial Hyls are classified as polysaccharide lyases, primarily within the PL8 and PL16 families. In contrast to mammalian Hyls, most bacterial Hyls are hyaluronate lyases, cleaving HA via a β-elimination mechanism rather than simple hydrolysis, resulting in the generation of unsaturated disaccharides (e.g., Δ4,5-uronic acid-GlcNAc) as the primary reaction product [2]. Microbial Hyls are characterized as Mn^2+^/Ni^2+^ sensitive enzymes with optimal activity for most of the Hyls at 37–45 °C and within a pH range of 5.5–7.0 [34,58]; however, metal ion sensitivity and optimal pH and temperature vary across species and enzyme classes. For example, Hyls from marine *Vibrio* species typically exhibit optimal activity at around 30 °C [59], whereas the recombinant Hyl from *Thermasporomyces composti* shows a much higher optimal temperature of approximately 70 °C [60], reflecting adaptation to distinct ecological niches. Regarding substrate specificity, most bacterial Hyls, excluding Hyl from *Streptomyces hyalurolyticus,* also act on chondroitin or chondroitin sulphate, with enzymatic activity influenced by the degree and pattern of sulfation [61,62,63].

Among microbial Hyls, bacterial Hyls are well characterized both structurally and functionally. Hyl production has been described in a variety of Gram-positive and Gram-negative bacteria, with notable distinctions in enzymatic activity, biological function, and subcellular localization [2,24,59,64]. Unlike Hyls from Gram-positive pathogens, which are secreted or covalently anchored to the cell wall via a conserved C-terminal LPxTG sorting motif, HA-degrading enzymes from Gram-negative bacteria are primarily localized in the periplasmic space [2,65,66]. Although Gram-negative bacteria can secrete Hyls extracellularly or display them on the outer membrane [59,65], initial depolymerization of HA is often followed by transport of HA fragments across the outer membrane via specific uptake systems into the periplasm, where these enzymes further cleave HA into unsaturated oligosaccharides [66,67]. In some species, such as *Vibrio alginolyticus* LWW-9, extracellular Hyls degrade HA into unsaturated disaccharides outside the cell, which are subsequently imported into the cytoplasm through a phosphotransferase system [59]. Collectively, these findings suggest that Gram-negative bacteria primarily exploit HA degradation as a nutrient acquisition strategy rather than as a direct mechanism for tissue invasion or virulence, in contrast to many Gram-positive pathogens, where Hyls play well-established roles in microbial pathogenesis [10,24]. Additionally, sequence-based phylogenetic analyses indicate that Hyls from Gram-positive bacteria, excluding *Clostridial* species, form a related group [2,24,68]. The Hyls from Gram-negative bacteria and *Clostridial* species are phylogenetically more divergent and lie outside the main Gram-positive Hyl clade. The classification and phylogeny of bacterial Hyls are further reviewed elsewhere [2,24,68], highlighting the evolutionary diversity within this enzyme family.

Hyls are also produced by several micromycetes and higher fungi [69,70]. Among higher fungi, the HA lyase from *Fistulina hepatica* DSM4987 was the first to be isolated and characterized; it degrades HA into unsaturated tetrasaccharides and exhibits optimal activity at approximately 20 °C [70]. In contrast, micromycetes such as *Talaromyces* species degrade HA optimally under acidic conditions (pH ~4.0) and at higher temperatures (~43 °C) [69]. Despite sharing low sequence homology with mammalian Hyls, micromycete enzymes display similar hydrolytic cleavage of β-1,4 glycosidic bonds in HA, generating saturated, even-numbered oligosaccharides with N-acetyl-D-glucosamine at the reducing end [69,70]. Notably, micromycete Hyls lack chondroitinase activity, and HA degradation ultimately yields saturated disaccharides as the final products [69,70].

Bacteriophages infecting *Streptococcus pyogenes* and *S. equi* also encode Hyls that degrade the HA capsule expressed by the bacteria, thereby facilitating phage access to the cell surface during infection [71,72,73]. Phage-encoded Hyls are generally shorter than their bacterial counterparts and share very low homology to bacterial Hyls. Despite all this, they cleave HA via the same catalytic β-elimination of the β-1,4 glycosidic bonds, yielding defined oligosaccharides, including tetra-, hexa-, octa-, and deca-saccharides [71,72]. Structurally, bacteriophage Hyls possess an extended substrate-binding region compared with bacterial Hyls, which is thought to contribute to their distinct product profiles [71,72].

## 3. Microbial Manipulation of Host HA for Colonization and Infection

Microbial exploitation of host HA represents an important evolutionary strategy to enhance host colonization and infection [10,24]. A study by Marion et al. demonstrated that *S. pneumoniae* efficiently utilizes HA-derived degradation products as a sole carbon source both in vitro and in vivo [74]. In chemically defined medium supplemented exclusively with HA, *S. pneumoniae* achieved maximal OD_600_ values comparable to those observed with glucose supplementation. Consistent with these findings, intranasal infection of mice with a Hyl-deficient *S. pneumoniae* mutant resulted in significantly reduced nasopharyngeal bacterial burden compared with the parental strain [74], highlighting the potential contribution of HA utilization to colonization and infection in vivo.

Comparable nutrient-scavenging mechanisms have been described in Group A *Streptococcus* (GAS) strains [75]. The GAS Hyl exhibits strict substrate specificity for HA, with no detectable activity against other ECM glycosaminoglycans such as chondroitin sulfate or heparan sulfate. Growth assays in minimal medium supplemented with individual HA-derived carbohydrates revealed that Hyl-positive GAS strains grow efficiently in the presence of N-acetylglucosamine (GlcNAc), but not glucuronic acid (GlcA) or chitotriose, a β-1,4-linked GlcNAc oligomer [75]. These observations indicate that GAS selectively exploits Hyl-mediated HA degradation to access GlcNAc as a preferred nutrient source, a strategy likely advantageous in nutrient-limited host environments encountered during dissemination and transmission. A similar HA-dependent growth phenotype has been reported in pathogenic Mycobacterium [76]. Collectively, these studies highlight HA degradation and utilization as a conserved metabolic strategy employed by diverse bacterial pathogens to support survival, persistence, and colonization within host tissues. However, not all bacteria exploit this mechanism for colonization or infection. In the case of *Enterococcus faecalis*, Hyl-mediated bladder colonization and bloodstream infection appear to be driven primarily by ECM remodeling rather than by the utilization of HA as a nutrient source [77].

**Figure 2:**
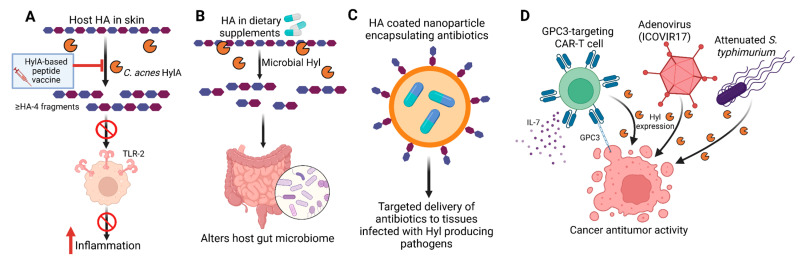
Therapeutic applications of Hyls. HA can be leveraged in multiple therapeutic contexts to modulate host physiology and enhance drug efficacy. (**A**) HylA-based peptide vaccine targeting *C. acnes* HylA activity, thereby preventing TLR2 stimulation and reducing inflammation. (**B**) Dietary HA modulates gut microbiota composition via Hyl-mediated metabolism, influencing host health. (**C**) Intracellular HA-coated nanoparticles enable targeted delivery of antibiotics to infected tissues, improving therapeutic efficacy. (**D**) HA-modulated CAR-T cells or microbial carriers utilize Hyl activity to enhance antitumor responses in cancer therapy. HA: hyaluronan; Hyl: hyaluronidase; HylA: *C. acnes* hyaluronidase A; GPC3: Glypican 3; CAR-T cell: chimeric antigen receptor T-cell. Created in BioRender. Nonoguchi, H. (2026) https://BioRender.com/1o4duc8 (accessed on 6 February 2026).

Beyond their role in nutrient acquisition, Hyls function as classic “spreading factors” that facilitate tissue invasion and pathogen dissemination (Figure 1). Enzymatic degradation of HA reduces ECM viscosity and increases tissue permeability, thereby promoting microbial spread through host tissues [78,79]. Early experimental evidence for this concept identified a “spreading factor” produced by invasive *Staphylococcus aureus*, which was later shown to be a Hyl. This factor significantly increased lesion size in a rabbit skin infection model and enhanced lesion expansion caused by co-infecting bacterial and viral pathogens [78,79]. Subsequent studies further established *S. aureus* Hyl as an important virulence determinant across multiple murine infection models [78,80,81], where it contributes to the early stages of disease progression by promoting tissue penetration and local dissemination. Support for the spreading-factor paradigm extends beyond bacterial pathogens. An oncolytic adenovirus engineered to express a Hyl (ICOVIR17) induces localized HA degradation within glioblastoma tumors, thereby enhancing viral dispersion throughout the tumor mass [82] (Figure 2). This increased intratumoral spread results in significant tumor regression and improved survival in mouse models, providing compelling evidence that HA degradation broadly facilitates biological dissemination within dense tissue environments. 

Pathogenic bacteria exploit the Hyl-HA axis to evade host immune responses, thereby promoting colonization and infection. We previously demonstrated that Group B *Streptococcus* (GBS) Hyl-mediated generation of HA disaccharides blocks TLR2 recognition, limits tissue damage, and facilitates bacterial persistence [10], whereas *Cutibacterium acnes (formerly Propionibacterium acnes)* Hyl activity can promote either commensal colonization or inflammatory disease depending on the type of Hyl-generated HA product [24]. These findings are supported by additional studies showing that, in pregnancy-associated bacterial infections, Hyl-derived HA disaccharides stimulate IL-10 production by uterine macrophages, suppressing pro-inflammatory responses and facilitating ascending infection [83]. Notably, Hyls do not always promote colonization and infection through HA degradation. *S. suis* can manipulate Hyl synthesis to modulate host immunity via HA-independent mechanisms [84]. In certain invasive strains, Hyl is proteolytically truncated into four fragments, abolishing its canonical enzymatic activity. One such fragment, HylS′, is secreted and binds complement component C3b, inhibiting C5 convertase formation and preventing membrane attack complex (MAC) assembly. This process reduces C3b deposition on the bacterial surface, impairs phagocytic recognition, and promotes immune evasion and tissue invasion. Consistent with this mechanism, infection with a HylS-deficient *S. suis* strain resulted in significantly reduced bacterial burdens in the spleen, blood, brain, and lungs within 6 h post-infection, accompanied by improved host survival compared with infection by the wild-type strain [84]. Collectively, these studies highlight the multifaceted strategies by which pathogenic bacteria manipulate HA and Hyl-associated functions to acquire nutrients, remodel host tissues, evade immune defenses, and facilitate dissemination during colonization and infection.

## 4. Structure and Regulation of Bacterial Hyls

Microbial Hyls are structurally diverse, often larger enzymes that lack homology to mammalian Hyls and function extracellularly as virulence factors [2,24]. We recently published the crystal structure of *C. acnes* Hyls and compared them with known structures of Hyls from other bacterial species, including *S. pneumoniae*, *S. agalactiae*, and *S. coelicolor* [24]. Structurally, *C. acnes* Hyls consist of an N-terminal α-domain and a C-terminal β-domain connected by a short linker. The active site is located within a long substrate-binding cleft at the interface of the two domains and is primarily formed by structural elements from the α-domain, with minor contributions from the β-domain. This cleft is highly conserved and contains a catalytic tetrad (Tyr–His–Arg–Glu) together with charged residues that facilitate substrate binding and product release [24]. Comparison of *C. acnes* Hyls with other bacterial Hyls shows that overall folding is conserved [24,85,86]. Notably, the substrate-binding cleft in *C. acnes* Hyls adopts an unusually open conformation compared to other bacterial Hyls [24,87], which may influence substrate accessibility and enzymatic activity. Despite extensive research, the catalytic mechanism of bacterial Hyls remains poorly defined. It is proposed that HA adopts a pre-reactive conformation upon binding the substrate cleft via electrostatic complementarity. The catalytic tetrad, together with conserved Asx (Asn/Asp) residue(s), acidifies the C5 carbon of glucuronic acid (GlcA), while the active-site His acts as a base to capture the C5 proton, triggering electronic rearrangement and the formation of the C4-C5 double bond, thereby generating 4,5-unsaturated oligosaccharides. Concurrently, Tyr residues donate protons to the glycosidic oxygen, leading to cleavage of the β-1,4 glycosidic bond and subsequent dissociation of the reaction products from the cleft [2,64,87].

Because microbes exploit Hyls to facilitate tissue invasion and establish either commensal colonization or pathogenic infection, microbial Hyl expression and activity are tightly regulated by networks that link metabolic status and substrate availability to gene expression. A study by Ibberson et al. (2014) demonstrated that *S. aureus* Hyl expression and activity are repressed by CodY, a global transcriptional regulator that links metabolic status and nutrient availability to virulence production [78]. Inactivation of CodY results in increased Hyl activity, indicating direct regulatory control. Similarly, in *S. pyogenes* and in *S. pneumoniae,* Hyl expression and activity are regulated in response to nutrient conditions through transcriptional regulators such as CodY and RegR (a LacI/GalR family repressor), respectively [88,89]. Similarly, host-derived cues, including exposure to ECM components, inflammatory environments, and host tissues, may influence Hyl regulation. For instance, in *Clostridium perfringens* strain, genes within the HA degradation cluster, including *hysA* encoding Hyl, are induced in the presence of HA and repressed in its absence, indicating substrate-dependent regulation of HA-degrading enzymes [90]. A study by Jung et al. (2017) demonstrated that host-derived IL-10, an anti-inflammatory cytokine, suppressed Hyl production during the resolution phase of inflammation [32]. We previously reported that acne-associated phylotypes exhibit a highly inflammatory phenotype, and that knockdown of the Hyl gene abolishes this inflammatory activity [24]. It would therefore be of interest to investigate how host-derived inflammatory cues modulate Hyl expression and activity in *C. acnes* and other clinically relevant pathogens. 

Niche adaptation further drives divergent regulation and functional outcomes of microbial Hyls. In encapsulated strains, Hyl activity is often attenuated or inactivated to preserve the HA capsule as a critical antiphagocytic virulence factor that allows adherence to host receptors such as CD44 (Figure 1), whereas non-encapsulated strains retain functional Hyls to facilitate tissue dissemination and nutrient acquisition in environments lacking capsule-mediated protection. This diversity is exemplified by GAS, in which molecular and biochemical analyses revealed that serotype M4 lacks the antiphagocytic HA capsule due to deletion of the ABC capsule biosynthesis operon but retains an active HA-degrading Hyl. In contrast, many encapsulated GAS serotypes harbor point mutations that render Hyl nonfunctional [91]. These findings indicate that evolutionary pressures within distinct host niches select for retention, inactivation, or loss of Hyl activity depending on whether HA degradation confers a fitness advantage. 

The complexity in bacterial Hyl function underscores the importance of characterizing not only which bacteria secrete Hyls and how their expression is regulated, but also the types of HA fragments they generate. Such insights are critical to determine whether HA-degrading bacteria promote inflammation, impede immune recognition, compromise tissue integrity, or support microbial growth. A comprehensive understanding of host-bacterial Hyl interactions could therefore reveal novel prophylactic or therapeutic strategies to combat diseases caused by colonizing or invading pathogenic bacteria. To exemplify this, the following section will discuss how *C. acnes* employs distinct Hyl variants to either maintain healthy skin or promote acne pathogenesis, and how targeted modulation of Hyl activity can improve disease outcomes.

## 5. *C. acnes* Hyls: Role in Health and Disease

*C. acnes* is a common skin commensal that has been widely studied for its role in acne pathogenesis. Both healthy individuals and those with acne-prone skin are robustly colonized by *C. acnes* phylotypes [92,93,94], indicating that pathogenicity is driven in large part by phylotype-specific traits rather than mere bacterial presence. We and others have identified genetic determinants within *C. acnes* that either support skin homeostasis or promote inflammatory acne [24,93,94]. Among these determinants, Hyls have emerged as key modulators of host–microbe interactions and immune responses. We recently demonstrated that *C. acnes* encodes two mutually exclusive Hyl variants, HylA and HylB, which differ in enzymatic activity, HA cleavage patterns, and biological effects [24]. Interestingly, individual strains encode either HylA or HylB, but not both [24], indicating functional specialization. Structurally, HylA and HylB share a conserved α/β fold but exhibit subtle yet functionally important differences in the substrate-binding cleft that influence HA positioning, cleavage patterns, and product release [24]. From an evolutionary perspective, HylB adopts fully processive exolytic mechanisms, resembling most bacterial Hyls, and efficiently degrades HA into disaccharides [10,24,85,95]. In contrast, HylA represents an intermediate evolutionary state, retaining non-processive endolytic activity while partially acquiring processive features, and is less efficient and generates larger oligosaccharides, including HA-4 and HA-6 [24,61]. These observations support a model in which ancestral non-processive enzymes gradually evolved toward more efficient HA degradation strategies, with functional specialization in shaping host interactions. 

Although bacterial Hyls share a highly conserved active site, they differ in the size of HA products generated, which could significantly impact host–microbe interactions and disease pathology. For instance, we previously demonstrated that administration of GBS Hyl or HA disaccharides attenuated lung inflammation and decreased pro-inflammatory cytokine levels relative to controls, thereby mitigating lung tissue damage. This protective effect is mediated, at least in part, by HA disaccharides generated by GBS Hyl, which interfere with host-bacteria interactions through TLR2 signaling [10]. In contrast, the role of *C. acnes* Hyls in acne pathogenesis and other *C. acnes-related* disease pathologies is poorly defined. Our work demonstrated that HylA is strongly associated with acne-associated phylotypes, whereas HylB is associated with healthy skin [24], suggesting the role of HylA in disease pathology. Previously, it was speculated that *C. acnes* Hyls may contribute to acne development by increasing the permeability of the follicular epithelium to free fatty acids and other irritants, thereby promoting the inflammatory phase [96]. Our study provided experimental evidence for how *C. acnes* employs two Hyl variants to shape host-immune interactions and influence acne pathogenesis. We demonstrated that HylA-generated HA fragments activate the host immune system in a TLR2-dependent manner, inducing pro-inflammatory cytokines, thereby exacerbating disease pathology in our murine acne model [24]. Notably, this inflammatory phenotype is governed by specific residues in HylA that influence both cleavage specificity and inflammatory potential. For instance, experimental site-directed mutagenesis introducing the S452G substitution in HylA shifted enzymatic cleavage toward HA-2 disaccharide production, thereby reducing the generation of pro-inflammatory HA fragments and reversing the inflammatory phenotype [24]. Our findings are indirectly supported by a human study showing increased Hyl production in inflammatory acne lesions [96], suggesting that our observations in mice may also be relevant to humans. Collectively, these findings illustrate how evolutionary specialization of bacterial enzymes directly shapes host–microbe interactions and disease outcomes, providing a strong foundation for precision-targeted acne therapies.

## 6. Therapeutic Approaches Based on Microbial Modulation of HA Activities

Given their diverse and context-dependent roles in human disease, Hyls have emerged as valuable tools for medical diagnostics and intervention, as well as promising therapeutic targets in a wide range of microbe-driven and inflammatory conditions. Both microbial and mammalian Hyls, and their HA degradation products, are increasingly being explored for applications spanning infection control, inflammation, microbiome modulation, drug delivery, and cancer therapy (Figure 2 and Table 1).

Clinical identification of pathogens—anti-Hyl antibodies are part of the serologic profile used to diagnose recent *GAS* infections and associated complications, including rheumatic fever and post-streptococcal glomerulonephritis [108]. While Hyl activity assays were historically employed for pathogen identification, their clinical utility has declined with advances in molecular diagnostics.

### 6.1. Targeting Microbial Hyls to Ameliorate Infections and Acne Vulgaris

Microbial Hyls contribute to key steps in the pathogenesis of Gram-positive bacterial infections, as discussed above. The relative conservation of amino acid sequences among Hyls from Gram-positive pathogens [24] suggests that catalytic sites could be targeted to develop cross-pathogen therapeutic strategies capable of limiting bacterial dissemination and disease progression, for example, in Gram-positive infections. In contrast to infectious diseases in which anti-inflammatory Hyl activity promotes pathogen persistence, therapeutic targeting of *C. acnes* Hyls in acne vulgaris aims to suppress pathogenic inflammation. However, inhibition of the pro-inflammatory *C. acnes* HylA is challenging because pro- and anti-inflammatory Hyls are expressed by different *C. acnes* strains share high amino acid sequence homology [24]. Vaccination with full-length recombinant HylA generates antibodies that cross-react with HylB expressed by health-associated *C. acnes* strains [24]. In a murine acne model, such cross-reactive antibodies attenuated the anti-inflammatory effects of HylB and exacerbated acne pathology. To overcome this limitation, a peptide-based vaccine incorporating HylA-specific sequences absent from HylB demonstrated efficacy in reducing acne pathology without cross-reactivity [24]. Similarly, a selective peptide inhibitor targeting the HylA active site, designed based on crystal structure analyses, effectively dampened pathogen-driven inflammation in vivo [24]. These findings were supported by a study demonstrating that Cath-HG, an antimicrobial peptide derived from the skin of the frog Hylarana guentheri, inhibited *C. acnes* biofilm formation in vitro and exerted anti-inflammatory effects in a mouse model, at least in part, through the suppression of key *C. acnes* enzymes, including lipases and Hyls [109].

### 6.2. General Immunosuppressive and Anti-Inflammatory Agents

Hyls produced by many major pathogens exert anti-inflammatory effects by degrading pro-inflammatory HMW-HA, while simultaneously generating PAMP-blocking HA disaccharides [10]. In murine models of lipopolysaccharide-induced acute lung injury, both GBS Hyl and HA disaccharides demonstrated strong therapeutic efficacy, as assessed by blinded histopathology and inflammatory cytokine profiling. These findings suggest that microbial Hyls and HA disaccharides may serve as promising therapeutics for inflammatory and autoimmune conditions in which HA plays a pathogenic role, including systemic lupus erythematosus, rheumatoid arthritis, cystic fibrosis, type I diabetes, multiple sclerosis, and inflammatory bowel disease [97]. Notably, the risk for allergic reaction from immune cross-reactivity is modest given the low level of amino acid sequence homology between microbial and human hyaluronidases, but repeated injection of hyaluronidases can lead to neutralizing antibody formation and limit therapeutic efficacy.

### 6.3. Modulation of the Host Microbiome

Breakdown of HA present in dietary supplements has been shown to alter gut microbiota composition, boosting *Faecalibacterium*, *Bacteroides*, and inflammation-combating bacteria *Bifidobacterium* in an in vitro study [98]. Although HA can promote the growth of a broad range of bacterial species, HA of a certain molecular weight range preferentially boosts the growth of certain species of bacteria. For example, 32 kDa HA preferentially enhanced the abundance of *Bacteroides*, whereas 1500 kDa HA regulated the growth of *Faecalibacterium* [99]. Importantly, orally administered high molecular HA is resistant to gastric and upper intestinal secretions and therefore maintains its microbiome-modulatory activity in the upper gastrointestinal tract [110]. In mice, oral gavage with 34 kDa HA also altered gut microbiome composition, notably increasing the abundance of the protective commensal *Akkermansia muciniphila* [100]. HA improved clearance of *Citrobacter rodentium* and associated colitis, an effect phenocopied by transplantation of HA-altered microbiota. Direct colonization with *A. muciniphila* enhanced goblet cell numbers, mucin production, and epithelial antimicrobial peptide induction, alleviating *C. rodentium*-induced bacterial colitis. Thus, HA or microbial Hyl activity may positively influence the gut microbiome in disease contexts. Careful manipulation of this axis could broaden the scope of human diseases impacted by gut microbiome composition. 

### 6.4. Drug Delivery

The ability of HA to form a bulky, hydrated structure in the ECM poses a formidable barrier to inflammatory cells and drugs alike [111,112]. Clinically, Hyls injected concurrently with other drugs significantly improved the spread of anesthetics, insulin, or ophthalmologic agents, enhancing their efficacy [101,102]. This effect is both dose-dependent and time-limited, with complete restoration of the barrier disruption by 48 h [102,113,114]. In a Phase III trial of 596 patients, subcutaneously administered trastuzumab with hyaluronidase was shown to be non-inferior when compared to IV trastuzumab in HER2-positive breast cancer, with comparable pathological complete response rates (45.4% vs. 40.7%) and equivalent long-term outcomes [115,116]. Conversely, HA is increasingly used as a filler to restore volume and enhance facial features [117]. Hyl addresses overfilling or complications in which the filler inadvertently blocks adjacent blood vessels [118]. Hyl also improves adverse effects associated with leaked fluid from intravenous lines by preventing swelling, inflammation, and necrosis caused by localized fluid pooling [119]. Specific to antibiotic delivery, several studies have shown that encapsulation of antibiotics in HA-based nanocoatings allows trigger release of the antibiotic payload in environments enriched with Hyl-expressing pathogens, such as *S. aureus* [103,104]. This approach provides more targeted delivery of antibiotics to infected tissue in the presence of Hyl-producing pathogens. HA-coated titanium nanotubes are also used to fabricate orthopedic implant materials that are more resistant to colonization by Hyl-secreting pathogens [120].

### 6.5. Tumor and Cell-Based Immunotherapies

Malignant tumors may be particularly amenable to adjunctive Hyl therapy due to frequent HA overexpression, which forms a physical barrier to immune cell infiltration and antitumor drug delivery [111,112]. Combining Hyl with chemotherapeutic agents has been shown to enhance intratumoral drug penetration and therapeutic efficacy. For example, expression of microbial Hyl in an attenuated *Salmonella typhimurium* strain enabled selective depletion of HA in pancreatic ductal adenocarcinoma, improving bacterial diffusion and antitumor activity following systemic administration [105]. Emerging strategies combining Hyl with CAR-T cell therapy have also demonstrated promising preclinical results. Xiong and colleagues engineered GPC3-targeting CAR-T cells to co-express IL-7 and PH20, resulting in robust antitumor activity in vitro and in vivo [106], suggesting potential for liver cancer therapy. Additional studies using biorthogonal approaches to conjugate α-PD-L1 antibodies and Hyl to CAR-T cells further improved antitumor efficacy in mouse models of B-cell lymphoma and colon cancer [107].

## 7. Conclusions and Future Directions

Microbial manipulation of HA in human diseases continues to be an active area of research. The field began with the recognition of HA as both a bacterial nutrient and a tool for host invasion, and has evolved with the discovery of how HA engages receptors that regulate numerous host physiological functions. The interaction of HA with TLR2/4 represents only a part of HA’s overall influence. Its broader physiological impact is mediated through interactions with other host receptors, which regulate tissue homeostasis, immune modulation, cell mobility, and HA clearance. The little-explored modulation of these pathways by microbial Hyls can add an additional layer of complexity to our understanding of how microbes influence host systems and contribute to human disease.

Despite the therapeutic potential of microbial Hyls, their clinical use requires careful consideration with respect to dosing and immunogenicity. Uncontrolled HA degradation can lead to tissue damage, inflammation, and increased susceptibility to infection. Advances in protein engineering, including the development of catalytically optimized or conditionally active enzymes, may help mitigate these risks. More research is needed to explore the combination of Hyls with advanced drug delivery systems, such as nanoparticles and cell-based therapies, to achieve spatially and temporally controlled ECM remodeling and targeted drug delivery. Mammalian Hyls offer physiologically compatible alternatives that are already well integrated into clinical practice. Comparative studies between microbial and mammalian Hyls will be essential to identify the most suitable enzyme sources for specific therapeutic indications. While most current medical applications of Hyls rely on bovine or ovine enzymes, microbial Hyls offer distinct advantages. Most microbial Hyls exhibit anti-inflammatory properties relative to mammalian enzymes due to the size of their HA degradation products and demonstrate low cross-reactivity with mammalian Hyls, making them attractive candidates for both cosmetic and medical applications. Many bacterial Hyls are also highly stable and amenable to large-scale industrial production. Together, microbial and mammalian Hyls could provide a rich and expanding toolkit for therapeutic intervention across a wide array of pathological conditions. Overall, the combined exploration of microbial and mammalian Hyls offers a powerful framework for future translational applications, enabling targeted ECM modulation, improved drug delivery, and innovative therapies for infection, inflammation, and cancer.

## Figures and Tables

**Table 1:** Emerging therapeutic applications modulating microbial HA activity.

Therapeutic Category	Specific Approaches	References
Infection and Inflammation Control	1. HylA-based peptide vaccine targeting disease-associated *C. acnes,* inhibiting pro-inflammatory response. 2. Selective peptide inhibitor targeting the HylA active site in disease-associated *C. acnes,* dampening inflammation. 3. Microbial Hyls and HA disaccharide-based treatment against inflammatory and autoimmune diseases.	[24,97]
Microbiome modulation	1. HA of distinct molecular weights in dietary supplements differentially modulate bacterial species. 2. HA can alter the gut microbiome, contributing to the alleviation of bacterial colitis.	[98,99,100]
Drug delivery	1. Hyls co-administered with other drugs improved the spread of anesthetics, insulin, or ophthalmologic agents. 2. Encapsulation of antibiotics in HA-based nanocoatings allows triggered release of the antibiotic payload in environments enriched with Hyl-expressing pathogens, such as *S. aureus*.	[101,102,103,104]
Cancer therapy	1. Expression of microbial Hyl in an attenuated *Salmonella typhimurium* strain enabled the selective depletion of HA in pancreatic ductal adenocarcinoma. 2. GPC3-targeting CAR-T cells co-express IL-7 and PH20 hyaluronidase resulting in robust antitumor activity. 3. An oncolytic adenovirus engineered to express a Hyl (ICOVIR17) induces localized HA degradation within glioblastoma tumors. 4. An α-PD-L1 antibody and Hyl conjugation to CAR-T cells further improved antitumor efficacy in B-cell lymphoma and colon cancer.	[82,105,106,107]

## Data Availability

No new data were created or analyzed in this study.
